# Supplementary material for: Antimicrobial Resistance in the Aconcagua River, Chile: Prevalence and Characterization of Resistant Bacteria in a Watershed Under High Anthropogenic Contamination Pressure
Source: Antibiotics (Basel). 2025 Jul 2;14(7):669. doi: 10.3390/antibiotics14070669 (PMC12291820; doi:10.3390/antibiotics14070669)
Supplement: Supplementary file 1 [file antibiotics-14-00669-s001.zip › antibiotics-3673932-supplementary.pdf]

Supplementary material

Supplementary Table S1: Primers and PCR conditions for amplification of carbapenemases

Gene	Primer Sequences (5'-3')	Amplicon Size (bp)	PCR conditions			Ref
blaKPC	F: CGTCTAGTTCTGCTGTCTTG<br>R: CTTGTCATCCTTGTTAGGCG	798	95 ºC<br>95 ºC<br>55 ºC<br>72 ºC<br>72 ºC	10 min<br>30 sec<br>30 sec<br>1 min<br>10 min	30 cycles	[49]
blaVIM	F: GGTGTTTGGTCGCATATCGC<br>R: CCATTCAGCCAGATCGGCATC	504	95 ºC<br>95 ºC<br>60 ºC<br>72 ºC<br>72 ºC	10 min<br>30 sec<br>30 sec<br>30 sec<br>10 min	30 cycles	[50]
blaNDM	F: GGTTTGGCGATCTGGTTTTC<br>R: CGGTGATATTGTCACTGGTGTGG	452	95 ºC<br>95 ºC<br>60 ºC<br>72 ºC<br>72 ºC	10 min<br>30 sec<br>30 sec<br>30 sec<br>10 min	30 cycles	[49]
blaIMP	F: GGAATAGAGTGGCTTAAYTCT<br>R: CCAACYACTASGTTATCT	188	95 ºC<br>95 ºC<br>55 ºC<br>72 ºC<br>72 ºC	10 min<br>30 sec<br>30 sec<br>15 min<br>10 min	30 cycles	[51]
